# Bacteraemia among severely malnourished children infected and uninfected with the human immunodeficiency virus-1 in Kampala, Uganda

**DOI:** 10.1186/1471-2334-6-160

**Published:** 2006-11-07

**Authors:** Hanifa Bachou, Thorkild Tylleskär, Deogratias H Kaddu-Mulindwa, James K Tumwine

**Affiliations:** 1 Department of Paediatrics and Child Health Makerere University Medical School P O Box 7072, Kampala Uganda; 2 Centre for International Health, University of Bergen, Norway; 3 Department of Microbiology Makerere University Medical School P O Box 7072, Kampala Uganda

## Abstract

**Background:**

To establish the magnitude of bacteraemia in severely malnourished children, and describe the types of bacteria and antimicrobial sensitivity by HIV status.

**Method:**

Isolates were recovered from 76 blood specimens. Antibiotic susceptibility tests were performed using commercial antibiotic disks and demographic and clinical findings were recorded.

**Results:**

Of the 450 children 63% were male; median age 17.0 months (inter quartile range, IQR 12–24) and 57% had oedema. 151 (36.7 %) of 411 tested HIV-positive; 76 (17.1%) of 445 blood specimens grew bacterial isolates; 58% were Gram negative – *S. typhimurium (27.6%) *and *S. enteriditis (11.8%)*. *Staph. aureus *(26.3%) and *Strep. pneumoniae *(13.2%) were the main Gram positive organisms. There was no difference in the risk of bacteraemia by HIV status, age < 24 months, male sex, or oedema, except for oral thrush (OR 2.3 CI 1.0–5.1) and hypoalbuminaemia (OR 3.5 CI 1.0–12.1). Isolates from severely immuno-suppressed children (CD4% <15%) were more likely to grow *Salmonella enteriditis *(OR 5.4; CI 1.6 – 17.4). The isolates were susceptible (≥ 80%) to ciprofloxacin, ceftriaxone and gentamicin; with low susceptibility to chlorampenicol, ampicillin (< 50%) and co-trimoxazole (<25%). Suspicion of bacteraemia had 95.9% sensitivity and 99.2% specificity. Among bacteraemic children, mortality was higher (43.5% vs 20.5%) in the HIV-positive; OR 3.0 (95%CI 1.0, 8.6).

**Conclusion:**

Bacteraemia affects 1 in every 6 severely malnourished children and carries high mortality especially among the HIV-positive. Given the high level of resistance to common antibiotics, there is need for clinical trials to determine the best combinations of antibiotics for management of bacteraemia in severely malnourished children.

## Background

Despite recent efforts aimed at reducing child mortality in resource poor countries, 11 million children under the age of five years die each year, mainly from infections and malnutrition [1]. Bacterial infections occur frequently in malnourished children and carry high case fatality. In Uganda, there is only one documented study of blood stream bacterial infection among severely malnourished children [2], and that study was carried out during the pre- HIV/AIDS era. Whether the HIV/AIDS pandemic has changed the pattern of bacterial infection and antimicrobial sensitivity is unknown. In Mulago, Uganda's national referral hospital, severe malnutrition has the highest case fatality compared to other paediatric illnesses, but the role of bacteraemia in this is not clear. The objective of the current study was to establish the magnitude of bacteraemia in severely malnourished children, and to describe the types of bacteria and antimicrobial sensitivity by HIV status.

## Methods

We enrolled 450 children with severe malnutrition defined as symmetrical oedema involving at least the feet or severe wasting (weight for height less than 3 SD) or both. The children were aged below 60 months and admitted to the paediatric wards of Mulago, Uganda's national referral and teaching hospital. Two hundred and twenty were enrolled in September – November 2003 and 230 children in September – December 2004. The caregivers of these children gave informed consent. Anthropometric measurements were taken according to the WHO standard techniques and compared with National Centre for Health Statistics (NCHS) reference population [3] Severe malnutrition was defined as weight-for-height (or length for children less than 2 years) of < -3 z-score and/or presence of oedema. Children with a length below 49 cm were excluded from the study as the NCHS reference does not provide reference values for these children. A medical officer collected the children's demographic and health characteristics using a clinical history, physical examination and laboratory examinations of blood and urine specimens and chest x-ray at admission.

### Blood culture and sensitivity

Blood specimens were obtained from 445 of the 450 children. Two millilitres of blood were drawn from a peripheral vein under aseptic condition after cleaning the skin with 70% alcohol and 2% tincture of iodine.

Each blood sample was inoculated into 2 culture bottles each containing 20 ml of Brain Heart Infusion Broth and incubated at 37°C for 24 hours after which bottles were observed for turbidity. From bottles showing turbidity, Gram's stain was done and inoculation made on to 7% sheep blood, chocolate and MacConkey agar, respectively. The plates were then incubated at 37°C for 18 – 24 hours. Blood and chocolate agar plates were incubated under carbon dioxide. Culture bottles that did not show turbidity were further incubated for up to 10 days. A total of 21/445 (4.7%) of the blood specimens grew contaminants.

Identification of culture isolates were done according to standard methods, for Gram negatives, AP120E was used followed by serological identification of salmonella species and coagulate test for *Staphylococcus aureus*, Optochin for *Streptococcus pneumoniae *and X and V factors for *Haemophilus influenzae*. The Kirby-Bauer diffusion method was used to test the susceptibility to the isolates on Muller-Hinton Agar-2 [4]. Commonly prescribed antibiotics were tested and graded as sensitive or resistant (Fa. Biomeriuex, France). Resistance was defined by the zone diameter below that given in standard operating procedure (NCCLS 2003). For example, all enterobacteriaceae are resistant to ampicillin when the zone diameter is less than 13 mm and considered sensitive when the zone diameter is greater than 17 mm.

#### HIV serology tests

Blood was taken in 5 ml EDTA vacutainer tubes (Becton Dickinson, Franklin lakes, NJ USA) every morning between 8–11 am by venipuncture and transported within 4 hours to the Uganda Virus Research Institute (UVRI) laboratory, Entebbe for serological testing. HIV testing was performed using a standard HIV algorithm of two enzyme-linked immunoassays (EIA) in parallel. Western blot, real time polymerase chain reaction (RT-PCR) was performed to confirm a positive EIA test for children below 18 months old and children with indeterminate results on EIA. Complete blood counts, including differential counts, were done using a Beckman Coulter counter.

### Ethical considerations

Informed consent for participation in the study, including HIV testing, was obtained from the care givers who received pre-test and post-test counselling from an experienced multilingual study counsellor. The study was approved by the Regional Committee for Medical Ethics, Bergen, Norway (REK Vest), Makerere University Faculty of Medicine Ethics and Research Committee, Mulago Hospital Ethics Committee and the Uganda National Council for Science and Technology.

### Statistics

The sample size was calculated using the formula by Kish [4]. Assuming a prevalence of bacteraemia among severely malnourished children to be 50%, also reported elsewhere [5] and a margin of error of 5% and 95% confidence, the minimum sample size for establishing the prevalence of bacteraemia was 380.

The statistical analysis was done using SPSS version 13 (SPSS Inc, Chicago, IL 60606, USA). For continuous variables medians were used to measure central tendency and inter quartile range (IQR) for the spread of the dependent variables. For categorical variables, proportions were compared using the chi-squared or Fisher's exact test where appropriate. The children were grouped by their gender (male, female), age groups in months (≤24 months and > 24 months; ≤12 months and > 12 months), presence of oedema, HIV test result (positive or negative) and blood culture result (bacteraemia or no bacteraemia). Logistic regression analysis was used to identify factors independently associated with bacteraemia.

Kaplan-Meier life tables and curves were used to determine survival functions and display data. Cox's proportional hazards model was used to compare survival with and without bacteraemia adjusted for age-group, sex, type of severe malnutrition and independent variables that had p values below 0.2 in the univariate analysis. Children were censored on the day of death. A 2-tailed p-value of < 0.05 was considered significant.

## Results

Of the 450 severely malnourished children studied, 62.4% were males and the median age was 17.0 months (IQR 12–24, ranges 4 – 60). More than half (56%) of the children presented with oedema, and there was no difference by sex; OR 1.02 (95% CI 0.7–1.5). Commonly diagnosed infections on admission included respiratory tract infections (positive x-ray findings) and diarrhoea. Seventy six (17.1%) of the 445 blood specimens cultured, grew bacterial isolates.

Although 36.7% of the children tested positive for HIV-1, there was no significant difference in the proportion of children who had bacteraemia by HIV status, age-group, sex or presence of oedema (Table 1). However, the proportion of children with oral thrush who had bacterial infection was twice that of the children without oral thrush. Only oral thrush and hypoalbuminaemia remained significant after adjusting for other factors in a multiple regression analysis.

**Table 1:** Characteristics, clinical and laboratory diagnosis and outcome of severely malnourished children below 60 months of age by presence or absence of blood stream infections, Mulago hospital, Uganda.

	Blood bacterial pathogens		Odds Ratio (95% Confidence interval)	
Characteristics	Present n/total (%)	Absent n/total (%)	Unadjusted	Adjusted

Age ≤ 24 months	59/353 (16.7)	17/92 (18.5)	0.89 (0.49–1.61)	0.78 (0.40–1.54)
Sex: male	48/279 (17.2)	28/166 (16.9)	1.02 (0.61–1.71)	0.99 (0.54–1.80)
Oedema	47/251 (18.7)	29/194 (14.9)	1.31 (0.79–2.18)	1.55 (0.81–2.96)
Hypothermia	3/24 (12.5)	21/327 (18.6)	0.62 (0.18–2.15)	
Oral thrush	15/54 (27.8)	61/391 (15.6)	2.08 (1.08–4.01)*	2.31 (1.04–5.11)*
Acute diarrhoea†	16/91 (17.6)	75/354 (21.2)	1.05 (0.57–1.92)	
Persistent diarrhoea††	15/81 (18.5)	66/364 (18.1)	1.13 (0.61–2.11)	
Respiratory infections	47/277 (17.0)	15/93 (16.1)	1.06 (0.56–2.01)	
Bacteruria	13/79 (16.5)	47/207 (22.7)	0.96 (0.49–1.89)	
Severe anaemia	2/56 (9.1)	20/253 (07.9)	2.3 (0.53–10.2)	
Malaria	5/36 (13.9)	71/406 (17.5)	0.76 (0.29–2.03)	
Hypokalaemia	38/178 (21.4)	38/262 (14.5)	1.60 (0.78–2.17)	1.44 (0.79–2.61)
Hypoalbuminaemia	71/367 (19.3)	5/73 (6.9)	3.26 (1.27–8.39)*	3.54 (1.04–12.1)*
Positive HIV status	30/149 (20.1)	39/259 (15.1)	0.42 (0.84–2.41)	1.67 (0.88–3.15)
CD4<15%	15/58 (13.8)	58/274 (21.2)	0.61 (0.27–1.36)	1.99 (0.76–5.22)

* p-value < 0.05, † frequent loose watery stools lasting ≤ 14 days, †† frequent loose watery stools lasting >14 days

Of the 76 blood specimens growing bacterial isolates, 44 (58%) had Gram negative organisms, predominantly *Salmonella species *and *E. coli *(table 2). Among the Gram positive organisms, *Staphylococcus aureus *and *Streptococcus pneumoniae *predominated, table 2. There was no significant difference in the types or subtypes of blood bacterial organisms by HIV status. However, blood specimens from severely immuno-suppressed children (CD4% <15%) were more likely to grow *Salmonella enteriditis *species than those from children with higher CD4% (OR 5.4, 95% CI 1.7–17), even after adjusting for HIV status (OR 9.0; 95% CI 1.7–48). Of the 12/55 (28%) HIV negative children with CD4% of <15%, nine (75%) were aged between 9–23 months.

**Table 2:** Number and percentages of bacterial pathogens isolated from blood specimens of severely malnourished children below 60 months of age at admission to, Mulago Hospital by HIV status

**Bacteria pathogens**	**HIV+ n/total (%)**	**HIV -ve n/total (%)**	**Odds ratio (95% CI)**
**Gram positive (Total = 27)**	14 (52)	13 (48)	1.31 (0.49 – 3.48)
*Staphylococcus*			
*Aureus (n = 13)*	8 (62)	5 (38)	
*Coagulase negative (n = 5)*	3 (60)	2 (40)	
*Streptococcus pneumoniae (n = 9)*	5 (56)	4 (44)	
**Gram negative (Total = 41)**	17 (42)	24 (58)	0.76 (0.29 – 2.03)
*Salmonella*			
*Enteriditis (n = 9)*	3 (33)	6 (67)	
*Typhimurium (n = 19)*	8 (42)	11 (58)	
*Typhi(n = 5)*	2 (40)	3 (60)	
*Escherichia coli(n = 6)*	3 (50)	3 (50)	
*Haemophilus. Influenzae (n = 2)*	1(50)	1 (50)	

Blood specimens from children with oral thrush were more likely to grow *Salmonella typhimurium *(14.3%, 5/35) than those from children with with no oral thrush (4.6%, 13/280); (OR, 3.4, CI 1.14 – 10).

### Isolates susceptibility to antibiotics

The bacterial isolates were mainly susceptible (≥ 80%) to ciprofloxacin, ceftriaxone, gentamicin and erythromycin. They had low susceptibility to chlorampenicol, ampicillin (< 50%) and co-trimoxazole (< 25%), table 3. Susceptibility to ceftazidime, ampicillin and chloramphenicol was significantly higher for bacterial pathogens isolated from HIV positive compared to the HIV negative (table 4). Intermediate resistance to specific antibiotics was also observed (2 to ceftriaxone, 1 to ceftaxidime, 3 to cefuroxime, 2 to ampicillin and 1 to chloramphenicol).

**Table 3:** Number and percentages of susceptibility of bacterial pathogen isolated from blood specimens of severely malnourished children below 60 months of age to selected antibiotics by HIV status.

	Staphylococcus		Streptococcus Pneumoniae	Salmonella			Escherichia Coli	Haemophilus
					
Antimicrobials	Aureus n/total (%)	CoN^† ^n/total (%)	n/total (%)	Typhi n/total (%)	Enteriditis n/total (%)	T. murium^‡ ^n/total (%)	n/total (%)	Influenza n/total (%)
	
Co-trimoxazole	4/17 (23)	0/3 (0)	0/11 (0)	1/5 (20)	2/9 (22)	6/21 (29)	0/6 (0)	0/2 (0)
Ampicillin	13/20 (65)	2/5 (40)	7/12 (58)	1/5 (20)	2/9 (22)	0/21 (0)	1/6 (17)	0/2 (0)
Augmentin*	12/14 (86)	2/3 (67)	10/11 (91)	3/5 (60)	2/8 (25)	4/21 (19)	1/6 (17)	1/2 (50)
Chloramphenicol	12/19 (63)	3/5 (60)	8/11 (73)	0/5 (0)	3/9 (33)	6/21 (29)	3/6 (50)	1/2 (50)
Cloxacillin	3/5 (60)	0/1 (0)	5/8 (62)	-	-	-	-	-
Cefuroxime	7/14 (50)	0/4 (0)	4/10 (40)	2/5 (40)	8/9 (89)	12/21 (57)	4/5 (80)	0/2 (0)
Ceftaxidime	9/15 (60)	1/3 (33)	5/6 (83)	1/5 (20)	0/2 (0)	4/7 (57)	1/4 (25)	1/1 (100)
Erythromycin	8/13 (61)	2/3 (67)	6/6 (100)	-	-	-	-	1/1 (100)
Gentamicin	15/17 (88)	4/5 (80)	3/7 (43)	4/5 (80)	9/9 (100)	16/21 (76)	5/6 (83)	1/2 (50)
Ceftriaxone	13/19 (68)	4/4 (100)	8/11 (73)	4/5 (80)	9/9 (100)	19/21 (91)	5/5 (100)	2/2 (100)
Ciprofloxacin	9/10 (90)	4/5 (80)	3/4 (75)	5/5 (100)	2/2 (100)	7/7 (100)	3/3 (100)	1/1 (100)

* Amoxicillin-clav, ^†^Coagulase Negative, ^‡^Typhimurium

**Table 4:** Number and percentages of susceptibility of bacterial pathogen isolated from blood specimens of severely malnourished children below 60 months of age to selected antibiotics by HIV status.

	HIV status		
		
Antibiotics	positive	negative	
	n/total (%)	n/total(%)	Odds ratio (95% CI)
Co-trimoxazole	5/24 (21)	9/39 (23)	0.90 (0.25 – 3.24)
Ampicillin	16/30 (53)	12/39 (31)	0.33 (0.12 – 0.93)
Augmentin	14/25 (56)	19/37 (51)	1.04 (0.38 – 2.87)
Chloramphenicol	16/29 (55)	14/39 (36)	0.35 (0.13 – 0.95)
Cloxacillin	2/4 (50)	4/7 (57)	1.33 (0.11 – 16.0)
Cefuroxime	16/21 (76)	24/37 (65)	0.88 (0.31 – 2.45)
Ceftazidime	13/18 (72)	8/17 (47)	0.21 (0.05 – 0.9)
Erythromycin	8/12 (67)	8/8 (100)	2.67 (0.24 – 30.1)
Gentamicin	21/27 (78)	29/36 (81)	1.09 (0.32 – 3.71)
Ceftriaxone	27/32 (84)	35/39 (90)	0.16 (0.02 – 1.42)
Ciprofloxacin	14/15 (93)	14/15 (93)	1.0 (0.06 – 17.6)

### Mortality

Although the mortality among the 76 children who had bacteraemia was higher (28.9% vs. 23.0%) than among the 369 without bacteraemia, the difference was not significant; OR 1.4 (95% CI 0.8–2.4), table 5.

**Table 5:** Number and percentages of outcome of severely malnourished children who had bacterial blood stream infections at admission to Mulago hospital, Uganda

	**Outcome status**				
		
	**Dead**		**Alive**		
**Organism**	**n**	**(%)**	**n**	**(%)**	**OR (95% CI)**
*Staphylococcus aureus*	3	(20)	12	(80)	1.8 (2.8 – 6.7)
*Coagulase negative*	1	(20)	4	(80)	1.7 (0.2 – 16)
*Streptococcus pneumoniae*	4	(40)	6	(60)	1.8 (0.5 – 07)
*Haemophilus influenza*	1	(50)	1	(50)	-
*Salmonella enteriditis*	1	(11)	8	(92)	3.6 (0.4 – 31)
*Salmonella typhi*	0	(0)	5	(100)	-
*Salmonella typhimurium*	6	(29)	15	(71)	1.0 (0.3 – 3.1)
*Escherichia coli*	6	(100)	0	(0)	4.4 (2.8 – 6.7)

**Figure 1:**
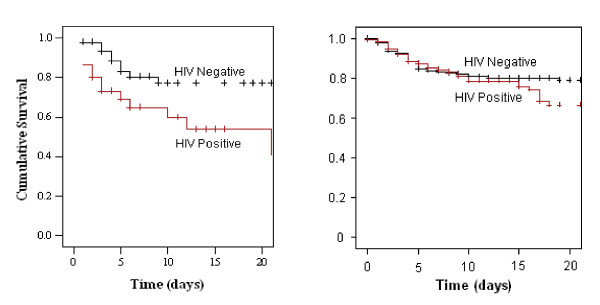
Kaplan-Meier survival curves of HIV positive and HIV negative children by blood culture positive and negative result.

Among the children with bacteraemia, mortality was higher (43.5% vs. 20.5%) in the HIV positive than the HIV negative; OR 3.0 (95% CI 1.01–8.6). This difference was still apparent when the survival of the children with bacteraemia was analyzed by the log rank test (p = 0.02). For the children without bacteraemia, the test of equality of survival distributions by HIV status was not significant (p = 0.07, figure 1).

## Discussion

In this study we report the pattern of bacteraemia amongst severely malnourished children in Mulago hospital, Uganda, where the prevalence of HIV infection among paediatric patients is high [7]. The prevalence of bacteraemia of 17% among the severely malnourished children in the current study is about the same as the 13% reported by Philipps and Wharton [2] in the same hospital in the pre-HIV/AIDS era, and comparable to the 18.7% recently reported from Nairobi by Nooran et al [8]. Nonetheless, a positive HIV test did not significantly increase the prevalence of bacteraemia. This is in keeping with several studies of bacteraemia in the HIV/AIDS era [8,9], but different from Berkeley's [10] study in which HIV and malnutrition were independent risk factors for bacteraemia. This difference might be due to the fact that the current study was carried out on only severely malnourished children with a high risk of dying.

Gram negative organisms, especially non typhoidal *salmonella species*, were the predominant cause of bacteraemia in severely malnourished children, supporting early results from Uganda [2] and recent studies from Kenya, Malawi and Ethiopia [8,11-13].

Although there was no difference in the types of bacterial organisms by HIV status, blood specimens from severely immuno-suppressed children were more likely to grow *Salmonella enteriditis*. The mechanism for this is not very clear and may include the difficulty in clearing salmonella infections from infected macrophages and weak immune system, HIV may predispose the host to infection with *Salmonella enteriditis *[14] and this, in turn, promotes the production of HIV in the macrophages of the gastrointestinal tract mucosal cells, thus completing a vicious cycle. Although some of the non typhoidal salmonella in the current study were unusual, they were all from the blood cultures and from patients residing in the slums of Kampala city.

We also found a high proportion of Gram positive organisms particularly *Staphylococcus aureus*. The reason for the predominance of *Staphylococcus aureus *in this series is not clear as there was no associated skin ulceration. It is possible that vitamin A deficiency in severely malnourished patients [15] might have contributed to this. Several studies have suggested that vitamin A deficiency predisposes to *Staphylococcus aureus *through phagocyte dysfunction and decreased complement activity [16]. In Uganda, the national health program includes Vitamin A supplementation twice a year for all the children below 5 years of age, from the age of 9 months. Vitamin A is also routinely given as treatment to all children suffering from measles, tuberculosis, severe malnutrition and severe pneumonia. Other possible risk factors that could have contributed to *Staphylococcus aureus *include concurrent viral infections, chronic lung diseases in HIV positive children and prior hospitalization. However, none of the children had been hospitalized two weeks prior to the study.

We found a very low proportion of *H. influenzae *infection in these children and this may be explained by the incorporation of HiB vaccine into the expanded programme on immunization (EPI) in Uganda from 2002.

Our study demonstrated high bacterial resistance to commonly used antibiotics such as co-trimoxazole, ampicillin and chloramphenicol among both HIV positive and HIV negative children. These findings raise great concern as ampicillin, in combination with gentamicin, is routinely given to all children admitted with severe malnutrition at Mulago hospital [17] However there was high susceptibility to ciprofloxacin, ceftriaxone and gentamicin regardless of HIV test status and this concurs with recent finding from Kenya [8].

Surprisingly, blood isolates from HIV infected children were more susceptible to ampicillin and chlorampenicol than those from HIV negative children. This finding is at odds with results from an Ethiopian study which reported that isolates recovered from HIV-positive patients were significantly resistant to many of the antibiotics tested when compared to the isolates from HIV-negative patients [18]. However a recent study from Thailand, of antimicrobial susceptibility tests of bacterial pathogens from blood cultures of HIV-infected patients, found that Salmonella species were highly sensitive to amoxicillin/clavulanate, gentamicin, and ciprofloxacin [19]. The difference in sensitivity patterns of salmonellae species may probably be attributed to difference in accessibility and use of antibiotics.

These results leave us in an important dilemma. The organisms exhibited very low in vitro susceptibility to one of the drugs (ampicillin) currently recommended in combination with gentamicin for the management of presumed bacteraemia in severely malnourished children. However they showed high susceptibility to gentamicin and ciprofloxacin. This calls for further studies to determine the most feasible combination of antibiotics for the management of bacteraemia in severely malnourished children in this setting. In conformity with other studies, our study did not find clinical signs or symptoms that could be reliably used to predict bacteraemia [9,20].

The mortality among severely malnourished children with bacteraemia of 28.9% was comparable to findings from other centres in sub-Saharan Africa [8,11,13]. Overall, there was no significant association between bacteraemia and mortality in this vulnerable group of children. However among the children with bacteraemia, mortality was much higher in the HIV-positive than among the HIV-negative, table 5. This underscores the importance of early diagnosis and use of efficacious antibiotics [21]. In the current study we did not observe any significant relationship between outcome and age of the children, which is consistent with results of a recent study from Kenya [22].

A limitation of this study is the lack of information on prior use of antimicrobials and previous history of hospitalization that may be associated with bacterial resistance and types of isolates. However, the information on underlying diseases was collected using the clinical history, physical examination laboratory tests and chest x-ray. We used isolates from two samples of children form two different years. However, the severely malnourished children from the two samples came from the same community served by the hospital and the same methodology was used at the same seasonal periods, the same hospital setting and the same laboratories. The study was conducted in only one hospital in the country and may not be representative of the larger Ugandan population.

Further more, the selection of specific seasons may have a bearing on the spectrum of pathogens identified as well as on the severity of malnutrition. The study was carried out between September and December, which is the peak season for severe malnutrition in Uganda. Unfortunately, it was not possible to determine the effect of seasonality on non-typhoidal salmonellae.

## Conclusion

Bacteraemia (both Gram negative and Gram positive organisms) affects one in every 6 severely malnourished children and carries high mortality especially among the HIV-positive children. Given the high level of resistance to commonly used antibiotics, there is need for clinical trials to determine the most feasible combination of antibiotics (cheapest most effective, given orally) for the management of bacteraemia in severely malnourished children in this setting.

## Competing interests

The author(s) declare that they have no competing interests.

## Authors' contributions

All authors participated in the design of the study, interpretation of the results, statistical analysis and writing the manuscript. HB supervised patient recruitment, follow-up and data collection. All authors read and approved the final manuscript.

## Pre-publication history

The pre-publication history for this paper can be accessed here:


